# (+)-[^18^F]Flubatine as a novel α4β2 nicotinic acetylcholine receptor PET ligand—results of the first-in-human brain imaging application in patients with β-amyloid PET-confirmed Alzheimer’s disease and healthy controls

**DOI:** 10.1007/s00259-020-05029-w

**Published:** 2020-09-16

**Authors:** Solveig Tiepolt, Georg-Alexander Becker, Stephan Wilke, Diego Cecchin, Michael Rullmann, Philipp M. Meyer, Henryk Barthel, Swen Hesse, Marianne Patt, Julia Luthardt, Gudrun Wagenknecht, Bernhard Sattler, Winnie Deuther-Conrad, Friedrich-Alexander Ludwig, Steffen Fischer, Hermann-Josef Gertz, René Smits, Alexander Hoepping, Jörg Steinbach, Peter Brust, Osama Sabri

**Affiliations:** 1 Department of Nuclear Medicine, University of Leipzig, Liebigstraße 18, 04103 Leipzig, Germany; 2 Department of Medicine, University-Hospital of Padova, Via Giustiniani 2, 35128 Padova, Italy; 3 Electronic Systems (ZEA-2), Central Institute for Engineering, Electronics and Analytics, Research Centre Juelich, Wilhelm-Johnen-Straße, 52428 Juelich, Germany; 4 Helmholtz-Zentrum Dresden-Rossendorf, Research Site Leipzig, Permoserstraße 15, 04318 Leipzig, Germany; 5 Department of Psychiatry, University of Leipzig, Semmelweisstraße 10, 04103 Leipzig, Germany; 6 ABX advanced biochemical compounds GmbH, Heinrich-Gläser-Straße 10, 01454 Radeberg, Germany; 7 Helmholtz-Zentrum Dresden-Rossendorf, Bautzener Landstr. 400, 01328 Dresden, Germany

**Keywords:** (+)-[^18^F]Flubatine [(+)-[^18^F]NCFHEB], PET, α4β2 nicotinic acetylcholine receptors, Human brain, Kinetic modeling

## Abstract

**Purposes:**

We present the first in-human brain PET imaging data of the new α4β2 nicotinic acetylcholine receptor (nAChR)–targeting radioligand (+)-[^18^F]Flubatine. Aims were to develop a kinetic modeling-based approach to quantify (+)-[^18^F]Flubatine and compare the data of healthy controls (HCs) and patients with Alzheimer’s disease (AD); to investigate the partial volume effect (PVE) on regional (+)-[^18^F]Flubatine binding; and whether (+)-[^18^F]Flubatine binding and cognitive test data respective β-amyloid radiotracer accumulation were correlated.

**Methods:**

We examined 11 HCs and 9 mild AD patients. All subjects underwent neuropsychological testing and [^11^C]PiB PET/MRI examination. (+)-[^18^F]Flubatine PET data were evaluated using full kinetic modeling and regional as well as voxel-based analyses.

**Results:**

With 270-min p.i., the unchanged parent compound amounted to 97 ± 2%. Adequate fits of the time-activity curves were obtained with the 1 tissue compartment model (1TCM). (+)-[^18^F]Flubatine distribution volume (binding) was significantly reduced in bilateral mesial temporal cortex in AD patients compared with HCs (right 10.6 ± 1.1 vs 11.6 ± 1.4, *p* = 0.049; left 11.0 ± 1.1 vs 12.2 ± 1.8, *p* = 0.046; one-sided *t* tests each). PVE correction increased not only (+)-[^18^F]Flubatine binding of approximately 15% but also standard deviation of 0.4–70%. Cognitive test data and (+)-[^18^F]Flubatine binding were significantly correlated in the left anterior cingulate, right posterior cingulate, and right parietal cortex (*r* > 0.5, *p* < 0.05 each). In AD patients, (+)-[^18^F]Flubatine binding and [^11^C]PiB standardized uptake value ratios were negatively correlated in several regions; whereas in HCs, a positive correlation between cortical (+)-[^18^F]Flubatine binding and [^11^C]PiB accumulation in the white matter was found. No adverse event related to (+)-[^18^F]Flubatine occurred.

**Conclusion:**

(+)-[^18^F]Flubatine is a safe and stable PET ligand. Full kinetic modeling can be realized by 1TCM without metabolite correction. (+)-[^18^F]Flubatine binding affinity was high enough to detect group differences. Of interest, correlation between white matter β-amyloid PET uptake and (+)-[^18^F]Flubatine binding indicated an association between white matter integrity and availability of α4β2 nAChRs. Overall, (+)-[^18^F]Flubatine showed favorable characteristics and has therefore the potential to serve as α4β2 nAChR–targeting PET ligand in further clinical trials.

**Electronic supplementary material:**

The online version of this article (10.1007/s00259-020-05029-w) contains supplementary material, which is available to authorized users.

## Introduction

The cerebral cholinergic system plays an important role for attention, cognition, and addiction, mainly by modulation of other neurotransmitter systems (e.g., dopaminergic system) [1]. In this context, the nicotinic acetylcholine receptors (nAChRs) are of particular interest. In the human brain, the α4β2 subtype is the most frequent nAChR subtype. First, in vivo estimations of the distribution of nAChRs were performed by Nordberg et al. [2] using the positron emission tomography (PET) tracer [^11^C]nicotine. As the retention of [^11^C]nicotine in the brain is co-determined by blood flow, blood brain barrier transport, and unspecific binding [3], a precise correlation of changes in [^11^C]nicotine accumulation with the nAChR availability was impossible. This problem was solved by 3-pyridylether derivatives (i.e., 2-[^18^F]FA-85380, 6-[^18^F]FA-85380, and 5-[^123^I]IA-85380). However, their slow kinetics hamper their application especially in a routine clinical setting [4]. Thus, new PET ligands with more favorable characteristics have been developed. These radioligands are all derivatives of homoepibatidine, epibatidine, or 3-pyridylether derivatives [4]. Results of first applications in humans were published for (−)-[^18^F]Flubatine, [^18^F]AZAN, and [^18^F]XTRA showing favorable data for dosimetry and image quality as well as fast kinetics [5–7]. However, [^18^F]AZAN and [^18^F]XTRA are significantly metabolized [7]; (−[^18^F]Flubatine on the other hand showed only a low amount of metabolites [5, 8, 9]. Due to inter-individual variability, the application of a reference region was often required to detect α4β2 nAChR differences between patients and healthy controls [9–15].

(+)-[^18^F]-Flubatine is the enantiomer of (−)-[^18^F]Flubatine. Preclinical data showed a higher binding affinity and a similar metabolism but a slower kinetics for (+)-[^18^F]Flubatine compared with (−)-[^18^F]Flubatine [16, 17]. Therefore, it was assumed that the analysis of cerebral (+)-[^18^F]Flubatine distribution works without application of a reference region.

It is known from neuropathological studies that an alteration of the cholinergic system with loss of α4β2 nAChRs occurs in patients with Alzheimer’s dementia (AD) [18, 19]. Several PET and SPECT studies using 2-[^18^F]FA-85380 or 5-[^123^I]IA-85380 revealed a reduction of α4β2 nAChRs in both AD and MCI subjects compared with HCs in various brain regions [9–13].

Primary aim of this study was to evaluate kinetic model–based approaches to quantify the dynamic (+)-[^18^F]Flubatine data and to compare the data of HCs with the data of patients with AD. The primary analysis was performed without the use of a reference region to prove that the (+)-[^18^F]Flubatine binding affinity was high enough to detect differences. Secondary aims of this study were to investigate (i) whether cortical atrophy affected the regional (+)-[^18^F]Flubatine binding, (ii) whether (+)-[^18^F]Flubatine binding was correlated with cognitive test data, and (iii) whether (+)-[^18^F]Flubatine showed a correlation to β-amyloid (Aβ) plaques.

## Methods and material

### Participants

All study participants were non-smokers, drug-free for any kind of centrally acting medication, and had no history of neurological or psychiatric illnesses except those with AD who were recruited as patient cohort. All subjects underwent a clinical assessment including a thorough neuropsychological testing. HCs were required to achieve a Clinical Dementia Rating (CDR) score of zero and psychometric test results within an interval of one standard deviation from the mean value (adjusted for age and education). Patients with mild to moderate AD were characterized by a progressive cognitive decline with DSM-IV criteria for dementia and probable Alzheimer’s disease according to the NINCDS-ADRDA criteria, furthermore a Mini Mental State Examination (MMSE) score between 20 and 26 and a CDR of 0.5 or 1.0. All subjects underwent a [^11^C]PiB PET/MRI examination on a simultaneous PET/MRI system (Biograph mMR, Siemens Healthcare, Erlangen, Germany). As the trials of Aβ-targeting PET tracers showed that only 63–66% of patients with the clinical diagnosis of probable AD were histopathologically Aβ plaque positive [20–22], we decided to implement Aβ PET imaging using [^11^C]Pittsburgh Compound B (PiB) in our screening procedure. All HCs had to be Aβ PET negative (Supplementary Figure 1), with the MRI not revealing any pathological findings. In the AD patients, a positive Aβ PET (Supplementary Figure 1) scan and a medial temporal lobe atrophy of Scheltens score ≥ 1 [23] were required. Overall, we recruited 30 participants (14 patients with clinical diagnosis of mild AD and 16 HCs). We had 9 drop-outs because of withdrawn consent (*N* = 2 HCs) and abnormal β-amyloid PET/MRI findings (4 patients with the clinical diagnosis of mild AD were β-amyloid PET negative, one HC was β-amyloid PET positive, another HC showed a large cerebral cyst, and a third HC an old cerebral hemorrhage). Furthermore, one patient had to be excluded because of severe motion artifacts in the brain scans. Thus, the final study population consisted of 9 AD patients and 11 HCs. The demographic data are summarized in Table 1.

**Table 1:** Demographic data of the study population

	Healthy controls	Patients with AD	*p*
Male/female	4/7	2/7	–
Age [years]	67 ± 4	67 ± 25	0.96
Apo E4 positivity (heterozygote/homozygote)^#^	2/0	4/1	n.s.
MTLA	0.5 ± 0.7	1.7 ± 0.7	0.001
MMSE score	30 ± 0.5	25 ± 1.4	< 0.001
DemTect score	15.8 ± 2.4	8.8 ± 3.1	< 0.001
A-K-T-PR* score	87.9 ± 20.9	62.5 ± 28.3	0.038

*A-K-T-PR* Alters-Konzentrations-Test, percentile ranks; *AD* Alzheimer’s disease; *MTLA* medial temporal lobe atrophy according to [23]; Values are given as mean value ± standard deviation

^#^In one patient with AD and one HC, the apolipoprotein (Apo) E4 status was not ascertained

*In one AD patient, the A-K-T-PR was not ascertained

### Neuropsychological testing

Global cognitive performance was tested using MMSE, CDR, and DemTect. Attention was measured by A-K-T test (*German:* “Alters-Konzentrations-Test”). All subtests of the Consortium to Establish a Registry for Alzheimer’s Disease (CERAD) battery were assessed including TMT-A as well as immediate and delayed memory tests of the Wechsler Memory Scale (WMS). Furthermore, the Geriatric Depression Scale (GDS) scores were acquired.

### PET and MR image acquisition and processing

The [^11^C]PiB PET/MRI examination included a 3-T brain MR imaging with the following sequences: standardized 3D T1w MPRAGE, T2w, T2*w and SWI in various planes (axial, coronal, sagittal), T2w 3D volumetric sequence (SPACE), and a DIXON sequence.

Dynamic (+)-[^18^F]Flubatine brain PET data were obtained with a stand-alone PET system (ECAT Exact HR+, CTI/Siemens, Knoxville, USA) in 3D mode following a slow intravenous injection of 287 ± 12 MBq (+)-[^18^F]Flubatine. The specific activity at the time of injection was 8.16 × 10^5^ ± 1.67 × 10^5^ GBq/mmol in HC subjects and 7.88 × 10^5^ ± 9.45 × 10^4^ GBq/mmol in AD patients (*p* = 0.657). The injected mass at the time of injection was in HCs 0.074 ± 0.013 μg and in AD patients 0.076 ± 0.087 μg (*p* = 0.679). Emission data consisted of one dynamic scan (0-–90-min p.i., 23 frames) and three subsequent scans (120-–150-min p.i., 180-–210-min p.i., 240—270-min p.i., 6 frames per scan). For attenuation correction, a 10-min ^68^Ge-transmission scan was performed prior to tracer application. Furthermore, all standard corrections as implemented by the vendor were applied (scatter, random events, radioactive decay, and dead time). Reconstruction of the PET data was performed by the ordered subset expectation maximization method with 10 iterations and 16 subsets into a voxel size of 2.6 × 2.6 × 2.4 mm.

Prior to kinetic analysis, the PET data were pre-processed using the PMOD software (PMOD technologies Ltd., Zürich, Switzerland, version 3.5). The individual MRI data were spatially reoriented onto a standard brain data set similar to the Talairach space. Thirty-six volumes of interest (VOIs) were manually drawn on three consecutive slices of the reoriented individual 3D T1w MPRAGE data (Supplementary Figure 2). The PET data was corrected for motion artifacts using Statistic Parametric Mapping 12 (SPM-Software; Wellcome Trust Centre for Neuroimaging, University College London) as described in detail elsewhere [9]. PMOD was used to co-register the dynamic PET data with the individual T1 MPRAGE MRI in order to create the time-activity curves (TACs) for the kinetic analysis.

### Blood sampling and plasma data analyses

Arterial blood samples were obtained from each subject. Thirty minutes before the radiotracer injection, a 10 mL sample was taken. Twelve to sixteen 2-mL samples were drawn in the first 3 min following injection and further samples until 270-min p.i.. All samples were centrifuged to separate blood plasma from the remnants. The radioactivity in plasma aliquots was measured using a gamma counter (COBRA, Packard Instrument Company, Meriden, CT, USA) and corrected for decay of F-18.

To estimate the non-metabolized parent fraction of the tracer, plasma was obtained from arterial blood samples and deproteinized by addition of acetonitrile (1:2) followed by centrifugation at 10,000 RPM (6000 ×*g*) for 10 min. The plasma samples were then analyzed by HPLC. The HPLC system used consisted of a gradient pump (P580, Dionex, Sunnyvale, CA), equipped with an UV and radioactivity detector (UV170D, Dionex, Sunnyvale, CA and GABI Star, Raytest, Straubenhardt, Germany, respectively), and a fraction sampling device (Foxy Junior, Isco, Lincoln, NE).

The amount of parent compound was determined by dividing the peak area corresponding to unchanged tracer by the total sum of all peak areas occurring in the chromatogram. For the late time points of sampling (i.e., 210- and 270-min p.i.), the eluate from the HPLC column was collected in 1.5 mL fractions by means of a fraction sampling device and the containing radioactivity was determined by gamma counting. The amount of parent compound was then calculated by dividing the sum of the activity contained within the fractions corresponding to the unchanged tracer by the sum of all fractions collected. The plasma-free fraction of tracer *f*_p_ was measured for each subject by ultrafiltration [8] using the 10-mL blood sample taken 30 min before the radiotracer injection.

### Kinetic data analysis and modeling

**Fig. 1:**
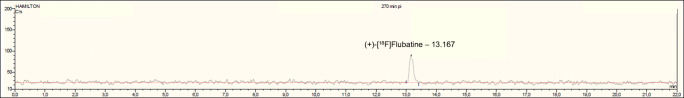
Metabolism of (+)-[^18^]Flubatine as measured in blood over time: over 97% unchanged parent compound at 270-min p.i.. The retention time (min) of a peak is given adjacent to the peak identification

1- and 2-tissue compartment models (TCM) were applied to analyze the time-activity curves (TAC). As the examination of the plasma probes did not reveal any relevant metabolites (Fig. 1), arterial input functions without metabolite correction were used. We applied the Akaike’s information criterion with correction for small sample sizes on the 90-min data of (+)-[^18^F]Flubatine [24, 25]. Model-based receptor parameters were the total distribution volume *V*_T_ (ml/cm^−3^), *V*_T_/*f*_p_ (ml/cm^−3^) and the distribution volume ratio (DVR) with the occipital cortex as reference region.

*V*_T_ is a region-dependent, linear function of the receptor density available for the tracer *B*_avail_, i.e., in case of the 2TCM

1 $$ {V}_{\mathrm{T}}=\frac{K_1}{k_2}\left(1+\frac{f_{\mathrm{ND}}{B}_{\mathrm{avail}}}{K_{\mathrm{D}}}\right) $$

where *K*_1_, *k*_2_ describe the transport into and out of the first (nondisplaceable) tissue compartment, *f*_ND_ the free fraction of tracer in the first tissue compartment, and *K*_D_ the dissociation constant of the receptor-ligand complex [26].

The distribution volume ratio DVR is under the assumptions that (1) the reference region is void of receptors, and (2) the nondisplaceable distribution volume *V*_ND_ = *K*_1_/*k*_2_ is the same in target and reference region, given by

2 $$ \mathrm{DVR}=\frac{V_{\mathrm{T}}}{V_{ND}}=1+\frac{f_{\mathrm{ND}}.\kern0.5em {B}_{\mathrm{avail}}}{K_{\mathrm{D}}}. $$

Further, Logan’s graphical analysis was applied to compute voxel-wise parametric images of *V*_T_/*f*_p_ in PMOD. Parametric images were spatially normalized in SPM12 by applying a non-linear transformation calculated based on the individual T1 MRI data. Smoothing was performed with an 8-mm Gaussian kernel.

### Reference region

(+)-[^18^F]Flubatine binding was quantified by the total distribution volume, a linear function of the local receptor density. Smaller (+)-[^18^F]Flubatine binding differences between AD patients and HCs might not reach significance because of inter-individual variability of nAChRs availability especially in a small sample size like ours. The implementation of a reference region is a usual approach to address this problem. As already noted, however, by definition, a reference region has to be void of receptors. Unfortunately, α4β2 nAChRs are ubiquitous in the human brain. Thus, we carefully considered to possibly use a “pseudoreference” region and chose for this purpose the bilateral occipital cortex, still being aware that this procedure has its limitations.

### Partial volume effect correction

To estimate the partial volume effect (PVE) on the (+)-[^18^F]Flubatine PET data, we additionally performed a region-based voxel-wise (RBV) partial volume effect correction as recently performed by our group for (−)-[^18^F]Flubatine PET data in AD and HCs (Sabri et al., 2018; Thomas et al., 2016; Thomas et al., 2011). Here, the PVE-corrected time-activity curves 0-–90-min p.i. were used for the kinetic analysis with a 1-tissue compartment model (1TCM) resulting in PVE-corrected distribution volumes (*V*_T_/*f*p_PVEC_).

### Determination of the cortical thickness

To investigate whether cortical thickness has an influence on the (+)-[^18^F]Flubatine PET data, the Desikan-Killiany cortical labelling protocol [27] was used. The isotropic (1 mm^3^) T1-weighted MR images were processed using FreeSurfer (v. 5.3) software (http://surfer.nmr.mgh.harvard.edu). After removing of the non-brain tissue and transformation onto the Talairach space, subcortical white matter and deep gray matter volumetric structures were segmented and normalized. Then, gray matter–white matter boundaries were calculated and parcellation of the cerebral cortex into units based on the gyral and sulcal structures was performed. The distance between the white matter and the pia mater was used as the thickness of each region [28].

### Statistical analyses

Statistical analyses were performed with MATLAB (version 7.13, The MathWorks, Natick, MA, USA) and IBM SPSS statistic software (version 24).

As a reduction of α4β2 nAChRs in AD patients can be assumed based on the results of our earlier studies [9, 10, 13] and autoradiographic findings, one-sided *t* tests were performed. The entire region set was used only for kinetic modeling. To compare (+)-[^18^F]Flubatine binding between AD patients and HCs, 10 regions (i.e., bilateral frontal, mesial temporal, parietal, anterior cingulate cortex (ACC), and posterior cingulate cortex (PCC)) were used. These candidate regions were chosen according to our previous results [9, 10, 13]. A correction for multiple testing was not applied. *p* < 0.05 was considered as significant [29], unless otherwise stated.

For correlation analysis between cortical thickness and *V*_T_/*f*_p_, we used partial correlations with age and sex as covariates. We correlated the cortical thickness and (+)-[^18^F]Flubatine binding (*V*_T_/*f*_p_) of the mesial temporal cortices and left precuneus as these regions showed significantly different (+)-[^18^F]Flubatine binding between AD patients and HCs. For this, the cortical thicknesses of the parahippocampal, entorhinal, fusiform, and inferior temporal regions of the Desikan-Killiany atlas were averaged and correlated with the *V*_T_/*f*_p_ values of the mesial temporal ROI.

Correlation analyses regarding cognitive test results and (+)-[^18^F]Flubatine binding were only performed for regions with significantly different (+)-[^18^F]Flubatine binding between AD patients and HCs. To consider age, sex, and educational differences, the raw data of the CERAD subtests were converted into *z* scores; and for the A-K-T data, the percentile ranks (PR) were used to consider age and gender differences. Thus, Pearson’s correlations without covariates were assessed between CERAD z scores and (+)-[^18^F]Flubatine binding (*V*_T_/*f*_p_) and partial correlations with education as covariate were calculated for correlation analyses between A-K-T-PR and *V*_T_/*f*_p_. For partial correlations between WMS, DemTect or GDS, and *V*_T_/*f*_p_, adjustment for age, sex, and education was carried out.

To correlate (+)-[^18^F]Flubatine binding and [^11^C]PiB uptake, seven regions (i.e., frontal, lateral temporal, parietal, occipital, anterior, and posterior cingulate cortex as well as white matter) were selected. These regions are established to investigate β-amyloid PET tracer uptake which is intense in cortical gray matter in AD patients and in white matter in HCs [30, 31].

For voxel-based comparisons performed using SPM 12, we used two-sample *t* tests with gender as covariate, *p* < 0.001 (uncorrected) as significance level, no global normalization, and *k* = 10 voxels as minimal cluster volume.

## Results

### (+)-[^18^F]Flubatine PET kinetic analyses and model selection

**Fig. 2:**
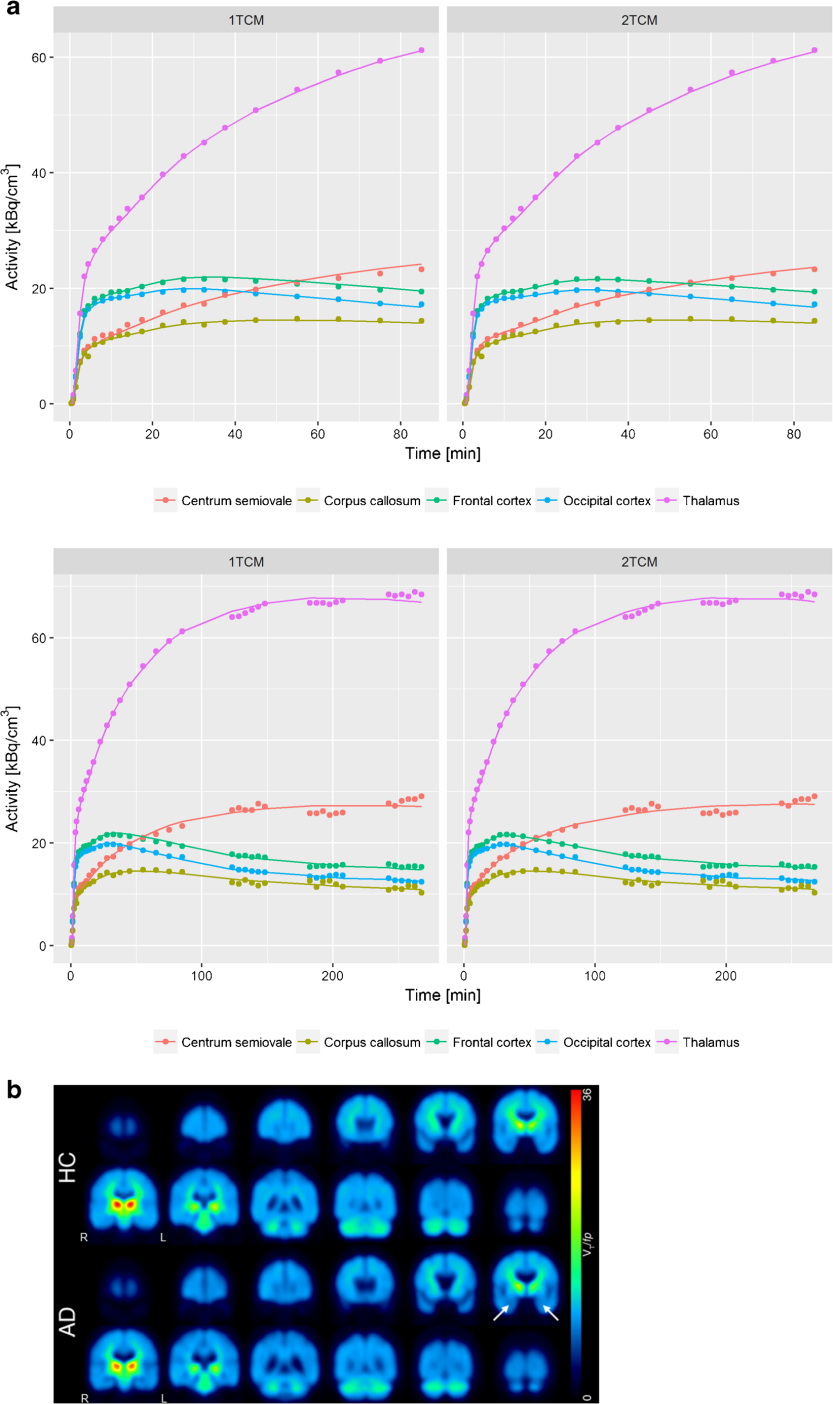
One-tissue compartment model (1TCM) and two-tissue compartment model (2TCM) applied to 5 brain regions of one HC (90- and 270-min data). PET activity measurements are given by points. Computed tracer concentrations in tissue are presented as lines (**a**). Mean parametric images (by Logan plots) of (+)-[^18^F]Flubatine (*V*_T_/*f*_p_) of the 11 healthy controls (HCs, top row) compared to the 9 patients with mild Alzheimer’s disease (AD, bottom row). White arrows indicate the significantly lower binding of (+)-[^18^F]Flubatine in the bilateral mesial temporal cortex in patients with AD (**b**)

The free fraction of (+)-[^18^F]Flubatine in plasma *f*_p_ was high and showed only small inter-individual variations (HCs 0.858 ± 0.020 (*n* = 11), ADs 0.869 ± 0.020 (*n* = 9)) with no differences between the groups (*p =* 0.251). The 1TCM and 2TCM could both be applied to describe the kinetics of (+)-[^18^F]Flubatine in brain tissue over time ranges of 0-–90-min p.i. to 0-–270-min p.i. and to compute receptor parameters. *V*_T_*/f*_p_ in all cortical regions could be reliably estimated from 0- to 90-min p.i. PET data. The Akaike information criterion (AIC) showed lower values for the 2TCM in the majority of HCs. The mean AIC values of the 11 HCs were in the right thalamus: 1TCM − 24.73 ± 10.19; 2TCM − 37.26 ± 16.82; in the left thalamus: 1TCM − 25.36 ± 10.14; 2TCM − 33.91 ± 16.75; in the right frontal cortex 1TCM − 31.47 ± 19.24; 2TCM − 61.02 ± 17.93; in the left frontal cortex: 1TCM − 34.02 ± 14.09; 2TCM − 59.98 ± 19.08; in the corpus callosum posterior: 1TCM − 39.51 ± 15.58; 2TCM − 49.58 ± 12.27. Thus, for (+)-[^18^F]Flubatine, the tracer kinetics could successfully be described with 1TCM (Table 2, Fig. 2). In the supplement, 1TCM and 2TCM estimations of all 36 brain regions and the corresponding *V*_T_*/f*_p_ and *K*_1_ values are presented (Supplementary Figure 3). Supplementary Figure 5 shows regional *V*_T_*/f*_p_ values of the a-priori regions, the α4β2 nAChRs rich thalami, and the corpus callosum posterior as region with lowest α4β2 nAChR density separately for HCs and patients with AD for different scan durations. Supplementary Table 1 summarizes the correlation analysis between regional *V*_T_/*f*_p_ values and injected mass in HCs for the a-priori regions.

**Table 2:** Quantitative (+)-[^18^F]Flubatine PET parameters in bilateral brain regions of the healthy controls

Brain region	0-–90-min p.i.		0-–270-min p.i.	
	*V*_T_*/f*_p_ (1TCM)	*V*_T_/*f*_p_ (2TCM)	*V*_T_*/f*_p_ (1TCM)	*V*_T_*/f*_p_ (2TCM
Frontal cortex	11.8 ± 1.7	12.0 ± 1.7	12.8 ± 1.7	12.6 ± 1.5
Lateral temporal cortex	11.9 ± 1.5	12.1 ± 1.5	12.6 ± 1.6	12.5 ± 1.5
Mesial temporal cortex	11.9 ± 1.5	12.1 ± 1.6	13.2 ± 1.8	13.2 ± 1.7
Parietal cortex	12.3 ± 1.3	12.6 ± 1.2	12.9 ± 1.8	12.7 ± 1.7
Occipital cortex	10.6 ± 1.3	11.0 ± 1.1	11.7 ± 1.3	11.6 ± 1.3
Anterior cingulate cortex	13.0 ± 1.8	13.4 ± 1.8	14.7 ± 2.5	14.3 ± 2.4
Posterior cingulate cortex	12.5 ± 1.8	12.9 ± 1.7	14.0 ± 2.2	13.7 ± 2.1
Caudate nucleus	14.2 ± 2.1	14.5 ± 2.0	16.1 ± 2.7	15.4 ± 2.4
Putamen	15.8 ± 2.5	16.7 ± 2.6	19.3 ± 3.2	18.3 ± 2.7
Thalamus	47.6 ± 11.3	47.8 ± 11.0	52.4 ± 13.0	48.3 ± 10.7
White matter	17.4 ± 3.4	20.3 ± 5.1	21.7 ± 5.7	21.2 ± 5.6
Pons/midbrain	17.6 ± 3.6	18.0 ± 3.7	19.1 ± 3.8	19.0 ± 3.7
Cerebellar cortex	14.9 ± 3.0	15.0 ± 2.9	16.0 ± 3.2	15.7 ± 3.0
Corpus callosum anterior	9.0 ± 1.3	9.5 ± 1.3	9.5 ± 1.4	9.6 ± 1.4
Corpus callosum posterior	8.9 ± 1.7	9.3 ± 1.6	9.4 ± 2.1	9.2 ± 1.9
Hypothalamus	19.9 ± 3.7	20.3 ± 3.5	22.6 ± 5.3	21.3 ± 4.7
Insula	13.3 ± 1.9	13.7 ± 1.6	15.0 ± 2.3	14.8 ± 2.2
Midbrain/substantia nigra	20.0 ± 3.8	20.7 ± 4.2	22.6 ± 4.6	22.4 ± 4.6
Hippocampus	12.1 ± 1.8	12.6 ± 1.7	14.1 ± 2.1	13.7 ± 1.8
Amygdala	11.6 ± 1.3	11.5 ± 1.3	11.9 ± 1.2	11.7 ± 1.2

*V*_T_*/f*_p_ total distribution volume divided by the free fraction in plasma; *1TCM* one-tissue compartment model, *2TCM* Two-tissue compartment model. Values are given as mean value ± standard deviation

### (+)-[^18^F]Flubatine rate constants *K*_1_ and *k*_2_ estimated by 1TCM

We found a significantly different influx rate *K*_1_ between HCs and AD patients in the bilateral mesial temporal cortex (right: HCs 0.29 ± 0.03, ADs 0.25 ± 0.04, *p* = 0.02; left: HCs 0.30 ± 0.04, ADs 0.26 ± 0.05, *p* = 0.03). Furthermore, we obtained significantly different washout constants *k*_2_ in the right frontal (HCs 0.035 ± 0.004, ADs 0.032 ± 0.004; *p* = 0.047), right mesial temporal (HCs 0.029 ± 0.003, ADs 0.027 ± 0.003; *p* = 0.035), and left mesial temporal cortex (HCs 0.029 ± 0.003, ADs 0.026 ± 0.003; *p* = 0.045) .

### (+)-[^18^F]Flubatine binding (*V*_T_/*f*_p_)

**Fig. 3:**
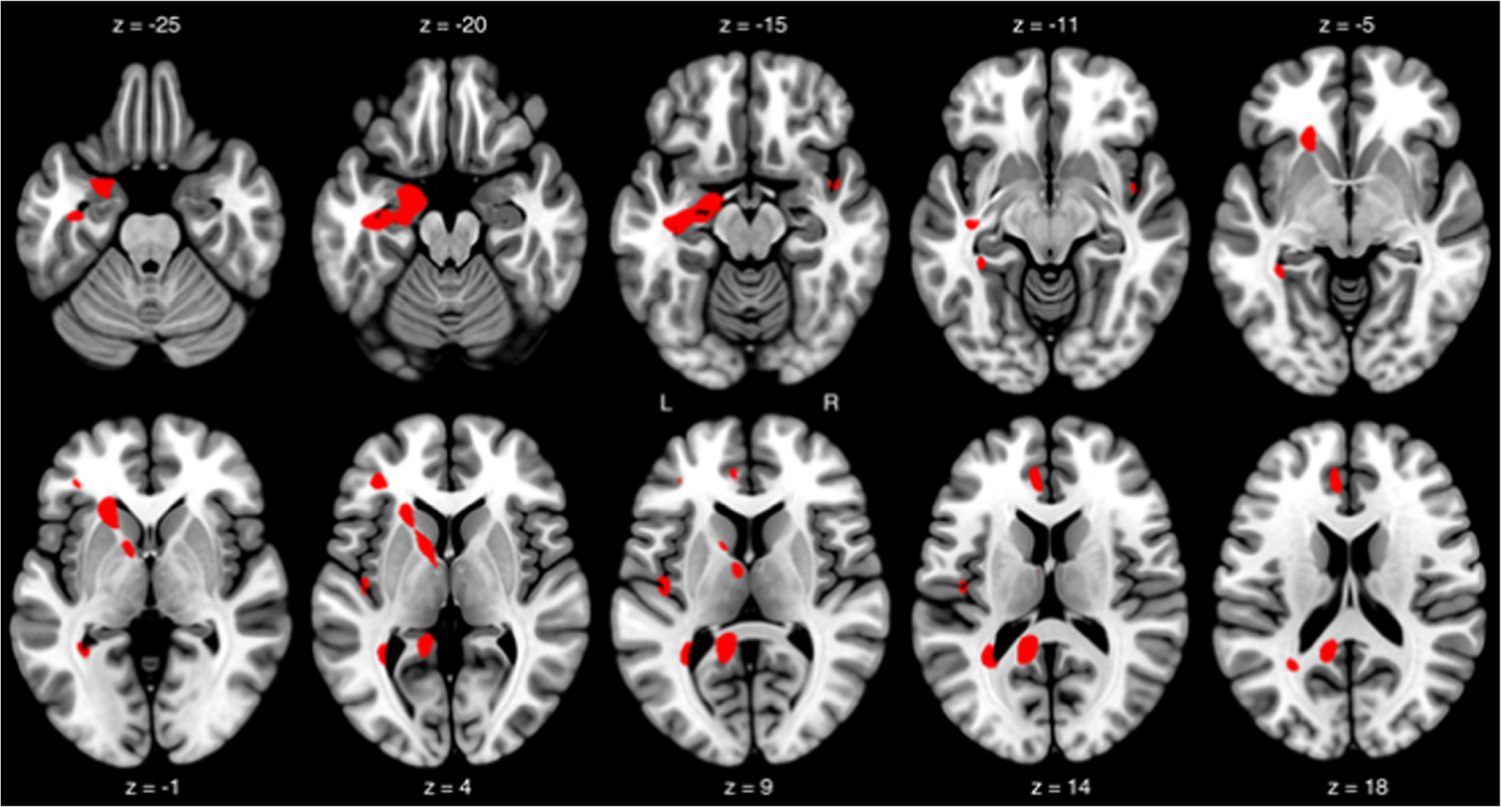
Voxel-based (SPM) analyses demonstrating the regions/clusters where Alzheimer’s disease (AD) patients had lower (+)-[^18^F]Flubatine binding (*V*_T_/*f*_p_) compared with healthy controls. *p* < 0.001 uncorrected, *k* > 30 voxels (covariable: gender)

**Fig. 4:**
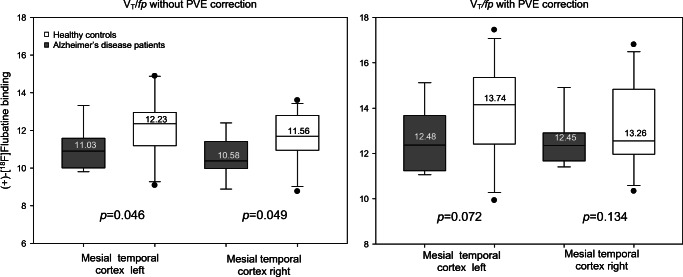
Boxplots depicting the (+)-[^18^F]Flubatine distribution volumes/free fraction in plasma (*V*_T_/*f*_p_) values in regions significantly different in the group analysis without partial volume effect (PVE) correction between the 9 patients with mild Alzheimer’s disease (AD) and the 10 healthy controls (HCs) and the *V*_T_/*f*_p_ values of the same regions after PVE correction. Numbers in the boxplots state the mean values

ROI-based analyses showed a significantly lower binding of (+)-[^18^F]Flubatine in the AD patients compared with the HCs in the mesial temporal cortices (Table 3, Fig. 3). Voxel-based analyses showed larger clusters (*k* > 100 voxels) of reduced (+)-[^18^F]Flubatine binding in AD patients in the left-sided hippocampus, precuneus, and putamen and smaller clusters (*k* = 30–100 voxels) in the right anterior orbitofrontal cortex, right paracentral lobule, left anterior cingulate cortex, and left triangular inferior frontal gyrus (Table 4, Fig. 4).

**Table 3:** Comparison of the regional (+)-[^18^F]Flubatine binding between patients with mild Alzheimer’s disease (ADs) and healthy controls (HCs)

Region	*V*_T_/*f*_p_		*p*
	HCs	ADs	
Left frontal cortex	11.8 ± 1.7	11.1 ± 0.94	0.134
Left mesial temporal cortex	12.2 ± 1.8	11.0 ± 1.1	0.046
Left parietal cortex	11.9 ± 1.3	11.5 ± 1.0	0.176
Left anterior cingulate cortex	13.0 ± 1.9	12.1 ± 1.3	0.113
Left posterior cingulate cortex	12.4 ± 1.9	12.1 ± 1.1	0.339
Right frontal cortex	11.8 ± 1.8	11.4 ± 0.9	0.247
Right mesial temporal cortex	11.6 ± 1.4	10.6 ± 1.1	0.049
Right parietal cortex	12.6 ± 1.6	11.7 ± 1.3	0.088
Right anterior cingulate cortex	12.9 ± 1.9	12.3 ± 1.2	0.188
Right posterior cingulate cortex	12.6 ± 1.8	12.1 ± 1.4	0.239

(+)-[^18^F]Flubatine binding is expressed as distribution volumes divided by the free fraction in plasma (*V*T/*f*p). *V*_T_ estimated are from 0- to 90-min p.i. PET scans. Values are given as mean value ± standard deviation

**Table 4:** Voxel-based analyses listing the regions/ clusters where patients with Alzheimer’s disease had lower (+)-Flubatine binding compared with healthy controls

Region	*k*	*p*_uncorr(cluster level)_	*p*_uncorr(peak level)_	*Z*	*x*, *y*, *z*
Left hippocampus	297	0.083	< 0.0005	4.04	−18 −10 −18
Left precuneus	238	0.117	< 0.0005	3.74	−12 −48 12
Left putamen	297	0.083	< 0.0005	3.64	−22 22 0
Left precuneus	224	0.127	< 0.0005	3.50	−30 −50 10
Right orbitofrontal cortex anterior	60	0.425	< 0.0005	3.72	24 72–22
Right paracentral lobule	31	0.576	< 0.0005	3.58	16–30 48
Left anterior cingulate cortex	71	0.384	< 0.0005	3.41	−6 36 16
Left triangular frontal inferior gyrus	40	0.520	< 0.0005	3.30	−38 38 4

(+)-Flubatine binding is expressed as *V*_T_/*f*_p_. *k* cluster size expressed in 2 × 2 × 2 mm voxels; *x*, *y*, *z* location of the peak in the three-dimensional stereotactic coordinates. Significance at *p* < 0.001 uncorrected (peak level), *k* > 10 voxels. Gender was used as covariate

### (+)-[^18^F]Flubatine binding using a reference region

Using the bilateral occipital cortex as reference region, the following regions showed significantly lower relative (+)-[^18^F]Flubatine DVRs in AD patients compared with HCs: right mesial temporal (1.02 ± 0.07 vs. 1.09 ± 0.06, *p* = 0.02), left mesial temporal (1.07 ± 0.05 vs. 1.15 ± 0.05, *p* = 0.001), and right parietal cortex (1.13 ± 0.07 vs. 1.19 ± 0.06, *p* = 0.03) and a further region showed a trend: left frontal cortex (1.08 ± 0.03 vs. 1.11 ± 0.06, *p* = 0.06), whereas left mesial temporal cortex survived correction for multiple testing.

### Partial volume effect correction of (+)-[^18^F]Flubatine PET data

Applying a PVE correction, the (+)-[^18^F]Flubatine binding (*V*_T_/*f*_p_) mean values increased by approximately 14.6% (range 7.4–22.1%) in the HC group and by approximately 17.1% (range 7.2–27.8%) in the AD group while the standard deviation increased by 24.4% (range 7.0–46.0%) and approximately 45.0% (range 0.4–69.5%), respectively. Mean PVE-corrected (+)-[^18^F]Flubatine binding values (*V*_T_/*f*p_PVEC_) of the right mesial temporal cortex were 13.26 ± 1.90 in HCs and 12.45 ± 1.08 in AD patients (*p* = 0.13) and of the left mesial temporal cortex 13.74 ± 2.07 in HCs and 12.48 ± 1.48 in AD patients (*p* = 0.07). Figure 4 and Supplementary Figure 6 illustrate the effect of the PVE correction on the mean *V*_T_/*f*_p_ values and standard deviation.

### Cortical thickness and (+)-[^18^F]Flubatine PET data

In 8 of 11 left-sided and 7 of 11 right-sided regions, the cortical thickness in the AD patients was significantly lower than in the HCs (Table 5). When correcting for age and gender, (+)-[^18^F]Flubatine binding (*V*_T_/*f*_p_) and cortical thickness were not correlated in the mesial temporal cortices: entire study population (left *r* = 0.18, *p* = 0.48; right *r* = 0.44, *p* = 0.07); HCs (left *r* = − 0.41, *p* = 0.28; right *r* = 0.42, *p* = 0.92); AD patients (left *r* = 0.64, *p* = 0.12; right *r* = 0.72, *p* = 0.07) nor in the left precuneus: entire study population (*r* = 0.23, *p* = 0.36); HCs (*r* = 0.1, *p* = 0.81); AD (*r* = 0.11, *p* = 0.82). Without covariates. a correlation between (+)-[^18^F]Flubatine binding (*V*_T_/*f*_p_) and cortical thickness were solely detectable in the left mesiotemporal cortex in the AD group (entire study population: left *r* = 0.29, *p* = 0.22; right *r* = 0.29, *p* = 0.21; HCs: left *r* = − 0.50, *p* = 0.12; right *r* = 0.26, *p* = 0.44; AD patients: left *r* = 0.74, *p* = 0.02; right *r* = 0.44, *p* = 0.23) and still no correlation was detectable in the left precuneus: entire study population (*r* = 0.38, *p* = 0.10); HCs (*r* = 0.04, *p* = 0.91); AD patients (*r* = 0.65, *p* = 0.06).

**Table 5:** Regional cortical thickness in patients with mild Alzheimer’s disease (AD) and healthy controls (HCs)

Region	Left hemisphere			Right hemisphere		
	HCs	ADs	*p*	HCs	ADs	*p*
Caudal anterior cingulate	2.81 ± 0.28	2.56 ± 0.38	0.115	2.49 ± 0.34	2.41 ± 0.27	0.576
Entorhinal	3.37 ± 0.35	2.75 ± 0.66	0.016	3.52 ± 0.37	2.97 ± 0.59	0.020
Fusiform	2.60 ± 0.12	2.34 ± 0.25	0.006	2.63 ± 0.14	2.24 ± 0.29	0.001
Inferior temporal	2.68 ± 0.17	2.46 ± 0.21	0.019	2.75 ± 0.12	2.53 ± 0.21	0.010
Isthmus cingulate	2.32 ± 0.21	2.06 ± 0.29	0.033	2.32 ± 0.23	1.90 ± 0.29	0.002
Medial orbitofrontal	2.28 ± 0.14	2.07 ± 0.22	0.014	2.27 ± 0.20	2.12 ± 0.19	0.111
Parahippocampal	2.71 ± 0.29	2.44 ± 0.44	0.126	2.57 ± 0.21	2.31 ± 0.36	0.062
Posterior cingulate	2.47 ± 0.16	2.16 ± 0.35	0.017	2.43 ± 0.16	2.14 ± 0.35	0.027
Precuneus	2.18 ± 0.18	1.91 ± 0.29	0.021	2.18 ± 0.14	1.94 ± 0.28	0.020
Superior frontal	2.61 ± 0.11	2.32 ± 0.29	0.007	2.58 ± 0.16	2.33 ± 0.25	0.015
Superior parietal	2.05 ± 0.13	1.89 ± 0.23	0.059	2.00 ± 0.10	1.87 ± 0.19	0.058

Values are mm, given as mean value ± standard deviation

### Neuropsychological testing and (+)-[^18^F]Flubatine PET data

**Fig. 5:**
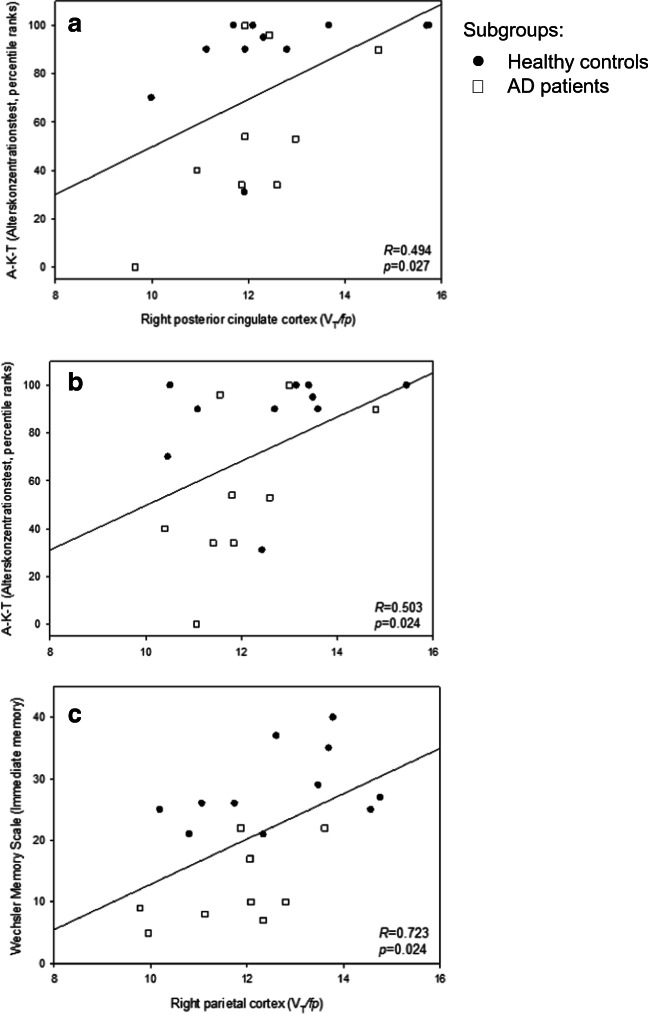
Graphs of the regression analyses showing **a** a significant linear regression between scores of the Alters-Konzentrations-Test (A-K-T) and (+)-[^18^F]Flubatine binding (*V*_T_/*f*_p_) of the left anterior cingulate cortex, **b** a significant linear regression between scores of the Alters-Konzentrations-Test (A-K-T) and (+)-[^18^F]Flubatine binding (*V*_T_/*f*_p_) of the right posterior cingulate cortex, and **c** a significant linear regression between scores of the Wechsler memory scale and (+)-[^18^F]Flubatine binding (*V*_T_/*f*_p_) of the right parietal cortex

The correlation analyses between z scores of the CERAD subtests (*n* = 11) and the regional (*n* = 16) (+)-[^18^F]Flubatine binding (*V*_T_/*f*_p_) did not reveal any significant correlations. In the entire study group performance in A-K-T-PR was significantly correlated with *V*_T_/*f*_p_ of the left anterior cingulate cortex (*r* = 0.504, *p* = 0.028) and right posterior cingulate cortex (*r* = 0.540, *p* = 0.017). Test data of WMS (immediate memory) were correlated with *V*_T_/*f*_p_ of the right parietal cortex (*r* = 0.541, *p* = 0.030). No correlations were found between regional *V*_T_/*f*_p_ and test scores of DemTect or WMS (delayed memory). Graphs of the regression analysis are shown in Fig. 5.

Examining the AD group alone performance in A-K-T-PR was not statistically correlated with *V*_T_/*f*_p_ of the anterior or posterior cingulate cortices, but a trend could be stated (left ACC *r* = 0.621, *p* = 0.074; right ACC *r* = 0.619, *p* = 0.075; left PCC *r* = 0.661, *p* = 0.052; right PCC *r* = 0.650, *p* = 0.058); test scores of WMS (immediate memory) and *V*_T_/*f*_p_ of the right parietal cortex were not correlated (*r* = 0.567, *p* = 0.112).

### [^11^C]PiB PET and (+)-[^18^F]Flubatine binding

**Fig. 6:**
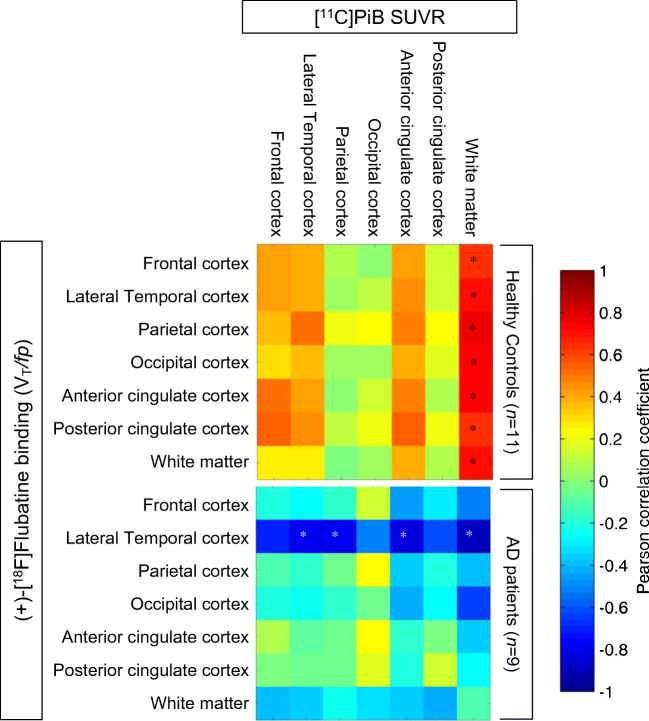
Matrices showing Pearson correlations between (+)-[^18^F]Flubatine binding (V_T_/*fp*) and [^11^C]PiB accumulation (standardized uptake value ratio (SUVRs), reference region: cerebellar cortex) across regions for the Alzheimer’s disease (AD) patients and healthy controls. Asterisks indicate significant correlations (*p* < 0.05)

The VOI-based analyses within the same region as well as across regions revealed significant correlations between (+)-[^18^F]Flubatine binding and [^11^C]PiB SUV ratios (reference region: cerebellar cortex) (Fig. 6). In the HCs, the white matter binding of [^11^C]PiB was positively correlated with the (+)-[^18^F]Flubatine binding in all cortical regions of interest (Fig. 6, Supplementary Figure 5). In the AD patients, (+)-[^18^F]Flubatine binding of the lateral temporal cortex was negatively correlated with [^11^C]PiB SUV ratios of the lateral temporal, parietal, and anterior cingulate cortex as well as the white matter (Fig. 6, Supplementary Figure 5).

### (+)-[^18^F]Flubatine PET safety evaluation

No serious adverse events occurred. None of the adverse events was related to the investigational product.

## Discussion

We present data of the first-in-human brain imaging study of the new α4β2 nAChR–targeting PET ligand (+)-[^18^F]Flubatine, the enantiomer of the recently introduced, highly α4β2 nAChR–specific (−)-[^18^F]Flubatine [5]. Overall, we found that (+)-[^18^F]Flubatine is a safe and stable radioligand with favorable characteristics. The correlation analysis between regional *V*_T_ values and injected mass in HCs did not show any significant correlation. However, in two of the 10 a-priori defined regions, the *p* values showed a trend towards significance. We assume that these two trends are spurious correlations as the receptor occupancy should be the same in all regions and therefore a violation of the tracer principle should be detectable by significantly correlations in the majority of the investigated brain regions. Furthermore, as mandatory in every subject, a safety factor of more than 1000 was ensured between the injected mass and the preclinical evaluated no observed effect level (NOEL) of 1.55 μg/kg. The visual evaluation showed that the dynamic PET data could be accurately analyzed with both a 1TCM and a 2TCM. Both approaches determined comparable *V*_T_/*f*_p_ values for all investigated brain regions. As the 1TCM approach is the more robust and the far less complicated matter, we decided to use this approach for the further analyses although the Akaike information criterion showed lower values for the 2TCM in the majority of the HCs. The fact that the distribution volumes can be sufficiently estimated with a 1 TCM could be an indication for a fast reversible binding of (+)-[^18^F]Flubatine to α4β2 nAChRs. As expected, the *V*_T_/*f*_p_ values of the homoepibatidine derivative (+)-[^18^F]Flubatine increased with α4β2 nAChR density known from post-mortem studies, especially [^3^H]epibatidine autoradiographic binding studies [32–34]. Thus, we found in accordance with these studies the highest (+)-[^18^F]Flubatine uptake in thalamus [32–34]. White matter, putamen, caudatus, pons, and substantia nigra showed an intermediate accumulation of (+)-[^18^F]Flubatine and cortical regions had the lowest *V*_T_/*f*_p_ values. These results are also in line with different autoradiographic binding studies using [^3^H]epibatidine or [^3^H]nicotine to quantify nAChRs [32–36]. Furthermore, this regional distribution pattern was observed in several previous PET studies using other α4β2 nAChRs targeting radioligands [5, 10–12, 15, 37]. The tracer kinetics in HCs and patients were similar and a scan duration of 90 min was sufficient to estimate the distribution volumes in all cortical regions in HCs as well as AD patients. Thus, the kinetics of (+)-[^18^F]Flubatine is faster than the kinetics of the early-generation α4β2 nAChR–targeting PET ligands like 2-[^18^F]FA-85380 [13]. The kinetics of (+)-[^18^F]Flubatine was moderately slower compared with that of the recently published (−)-[^18^F]Flubatine, but faster than assumed by preclinical data [16]. In contrast to (−)-[^18^F]Flubatine which showed a low number of metabolites [5, 8, 9], metabolic degradation of (+)-[^18^F]Flubatine was negligible. Therefore, a metabolite correction for (+)-[^18^F]Flubatine is not required at all. We compared (+)-[^18^F]Flubatine binding in 9 patients with mild to moderate AD with that in 11 healthy controls. Using voxel-based analysis, we obtained significantly reduced α4β2 nAChR availability in the bilateral mesial temporal cortex, in the left precuneus, left putamen, and in smaller clusters also in the bilateral frontal cortex and the left anterior cingulate cortex in AD patients. In other regions, (+)-[^18^F]Flubatine binding differences between AD patients and HCs did not reach significance, potentially because of the inter-individual variability of the α4β2 nAChR availability. To minimize this variability in evaluating PET and SPECT images, the implementation of a reference region is typical. In former studies, we used the corpus callosum as reference region [5, 9, 10, 13]. However, this region is difficult to delineate and in a recently published study, (−)-[^18^F]Flubatine was displaceable by 21 ± 9% in the corpus callosum in smokers [38], indicating that at least in smokers, a relevant specific binding is available in this region which is known as the region with the lowest amount of α4β2 nAChRs in humans. Thus, the corpus callosum does not seem to be very suitable as reference region for (+)-[^18^F]Flubatine. As α4β2 nAChRs are ubiquitous in the human brain, an optimal reference region without receptor binding does not exist. Our regions of interest are gray matter regions. Therefore, the reference region should also consist of gray matter and should be large enough to be easily delineated. Thus, we determined the occipital cortices as most suitable reference region considering that this region is only affected in severe stages of AD [39]. The evaluation with this reference region showed relatively reduced availability of α4β2 nAChRs in the AD patients compared with HCs in the afore-noted mesial temporal cortices, and further in the right parietal cortex. A tendency in the same direction was also detected in the left frontal cortex. As all 10 candidate regions of interest were considered to be of substantive interest, a-priori (based on our results from earlier PET studies of nicotine receptors in AD patients with early-generation radiotracers [9, 10, 13]) no multiplicity adjustment was incorporated. However, the significances would not survive a correction due to multiple testing. Autoradiographic binding studies consistently found reduced binding sites in temporal and frontal cortices in patients with Alzheimer’s disease/dementia with reductions of approximately 50–60%, especially in the temporal cortices [19, 36, 40–46]. Furthermore, several studies using 2-[^18^F]fluoro-A-85380 as PET ligand revealed similar reduction patterns with reduced α4β2 nAChRs in the frontal, temporal, and anterior cingulate and posterior cingulate cortices as well as in subcortical regions (i.e., caudate, thalamus) [4, 9, 10, 12, 13, 15]. In the current study, the extent of the α4β2 nAChR reduction is comparatively small. The mild manifestation of the AD (mean MMSE score 25) might explain these results. Applying partial volume effect correction increased the detected values of (+)-[^18^F]Flubatine binding (*V*_T_/*f*_p_) by approximately 15%. The relation between the mean values of the HC and AD group remained unchanged. However, the standard deviation was also increased but by a larger and more inhomogeneous amount (0.4–70%). We observed very similar results using PVE correction in another recently published study of the enantiomer (−)-[^18^F]Flubatine [9]. Our data indicate that PVE correction might correct the binding values but simultaneously increases the variance and thus the statistical noise which itself impairs the group statistics, especially in small study cohorts, and results in loss of statistical significance. Hence, we used a second approach, i.e., measuring the cortical thickness to investigate whether cortical atrophy is the reason for reduced regional (+)-[^18^F]Flubatine binding in AD. Quantitatively, the cortical thickness in the AD patients, compared with healthy controls, was not only lower in the mesial temporal cortex and adjoining areas but also in most of the examined cortical regions. As expected [47], the bilateral entorhinal cortices and the isthmus of the left cingulate cortex showed the most severe atrophy with approximately 15–18% reduction in AD patients compared with HCs. To investigate whether cortical atrophy and (+)-[^18^F]Flubatine binding were associated, we correlated the values of the mesial temporal cortices and the left precuneus where the largest clusters of lower (+)-[^18^F]Flubatine binding were located. These data were not correlated using age and gender as covariates. Without covariates, we revealed a correlation between (+)-[^18^F]Flubatine binding and cortical thickness solely in the left mesiotemporal in the AD patient. Therefore, we assume that the different α4β2 nAChR availability between AD patients and HCs in the left precuneus and in the right mesiotemporal cortex was not significantly affected by cortical atrophy. However, the different 4β2 nAChR availability in the left mesiotemporal cortex might partially be caused by cortical atrophy in the AD patients. Furthermore, we found a reduced influx and washout constant in the bilateral mesial temporal cortices in the AD patients. The estimation of regional *V*_T_ values by correct kinetic modeling is independent of perfusion differences between the study groups and therefore (+)-[^18^F]Flubatine *V*_T_ values reflect the availability of α4β2 nAChR alone. However, it cannot be completely ruled out that the observed reduction of uncorrected mesiotemporal (+)-[^18^F]Flubatine binding in the AD patients was driven by atrophy. The thorough neuropsychological testing revealed only three correlations between neurocognitive test data and (+)-[^18^F]Flubatine binding. This might be a result of the small number of patients who were, further, only mildly impaired (four patients had an MMSE score of 26 and further four of 24 or 25). The scores of the Alters-Konzentrations-Test (A-K-T) were correlated to the α4β2 nAChR availability in the left ACC and right PCC and immediate memory scores (WMS) to the right parietal cortex. In other study populations with moderately impaired AD, patients cognitive test data and α4β2 nAChR availability were significantly correlated in several cortical regions especially regions that are typically affected in AD (i.e., frontal, temporal, parietal, and cingulate cortices) [9, 10, 13, 48].

As a screening procedure, all subjects underwent a β-amyloid simultaneous PET/MRI examination. Thus, we were able to also investigate the association between regional availability of α4β2 nAChR and β-amyloid accumulation. Okada et al. [12] correlated [^11^C]PiB (nondisplaceable binding potential-BP_ND_) determined in 5-mm VOIs in the medial prefrontal cortices to 2-[^18^F]FA-85380 (BP_RI_) of nine regions defined by a MRI atlas and found a negative correlation between [^11^C]PiB (BP_ND_) of the medial prefrontal VOI and 2-[^18^F]FA-85380 (BP_RI_) in the medial frontal cortex and nucleus basalis of Meynert. In contrast to Okada et al. [12], we used manually drawn VOIs and used those to determine and correlate the [^11^C]PiB and (+)-[^18^F]Flubatine binding. In our HC group, we obtained a positive correlation between [^11^C]PiB binding in the white matter and α4β2 nAChR availability of all investigated cortical regions. It has been demonstrated that [^11^C]PiB binding in the white matter is associated with myelin sheaths [31]. A higher density of myelin sheathed fibers should be associated with a higher density of neurons, receptors, and transporters which would result in a higher (+)-[^18^F]Flubatine binding. Another explanation for the positive correlation could be an indirect association between axonal transport in white matter and receptor availability. In AD patients, this positive correlation between white matter binding of [^11^C]PiB and α4β2 nAChR availability measured by (+)-[^18^F]Flubatine binding was not detected. This might be an expression of infirmity and/or impairment of these processes. Instead, we found a negative correlation between (+)-[^18^F]Flubatine binding of the lateral temporal cortex and [^11^C]PiB accumulation of several cortical regions (i.e., lateral temporal, parietal, and anterior cingulate cortex as well as white matter) in the AD patients. However, we did not find a correlation in the frontal region as published by Okada et al. [12]. Different analysis approaches (voxel vs. region based), radioligands (2-[^18^F]FA-85380 (BP_RI_) vs. (+)-[^18^F]Flubatine (*V*_T_/*f*_p_)), and modeling (e.g., with or without using a reference region) as well as study populations (20 moderate vs. 9 mild AD patients) might be explanations for the different results. The lateral temporal cortex receives its cholinergic fibers from the lateral pathway that travels ventrally from the nucleus basalis of Meynert directly in the temporal regions [49]. β-Amyloid deposits located in the lateral temporal cortex might impair these fibers which could explain the observed negative correlation between α4β2 nAChR availability and β-amyloid deposits in this region. However, a direct impairment of these fibers by β-amyloid deposits in the parietal or anterior cingulate cortex is unlikely. These negative correlations could be based on an indirect association. Examinations in larger study cohorts are necessary to verify these interesting results.

Overall, (+)-[^18^F]Flubatine as α4β2 nAChR–targeting PET radiotracer has the potential to be applied in further clinical trials examining neurological or psychiatric diseases like dementia, parkinsonian syndromes, or depression. Naturally, tobacco smoke or central acting medication with effect on α4β2 nAChR interferes with the cerebral accumulation of (+)-[^18^F]Flubatine like every other α4β2 nAChR–targeting radiotracer. The sufficient scan duration of 90 min is a substantial progress in comparison with the early-generation α4β2 nAChR–targeting PET ligands like 2-[^18^F]FA-85380. However, full kinetic modeling is still required until follow-up studies prove a simplified method as applicable. As noted already, a limitation of this study is the small sample size. The mild manifestation of AD in our patients was necessary to investigate whether reduced α4β2 nAChR availability is already detectable in this early clinical stage of disease. However, results of secondary aims, e.g., correlation between α4β2 nAChR availability and cognitive test data, might be less distinctive because of the coincidence of small sample size and mild manifestation of AD.

## Conclusion

(+)-[^18^F]Flubatine is a safe and stable PET ligand. The α4β2 nAChR availability can be quantified using (+)-[^18^F]Flubatine PET by a 1TCM. The amount of metabolites is negligible and therefore no metabolite correction needs to be applied to the arterial input function. A scan duration of 90 min is sufficient to analyze all cortical regions. The high (+)-[^18^F]Flubatine binding in subcortical structures is favorable. In comparison with HCs, patients with mild to moderate AD showed a reduced availability of α4β2 nAChRs in the bilateral mesial temporal cortex. Here, (+)-[^18^F]Flubatine binding affinity was high enough to distinguish groups without using a reference region. However, the use of a reference region increased the sensitivity. Of interest, correlation between white matter β-amyloid PET uptake and (+)-[^18^F]Flubatine binding indicates an association between white matter integrity and availability of α4β2 nAChRs. Overall, (+)-[^18^F]Flubatine has the potential to serve as α4β2-targeting PET ligand in humans.

## Electronic supplementary material

ESM 1 (DOCX 6418 kb)
