# Supplementary material for: (+)-[18F]Flubatine as a novel α4β2 nicotinic acetylcholine receptor PET ligand—results of the first-in-human brain imaging application in patients with β-amyloid PET-confirmed Alzheimer’s disease and healthy controls
Source: Eur J Nucl Med Mol Imaging. 2020 Sep 16;48(3):731–46. doi: 10.1007/s00259-020-05029-w (PMC8036219; doi:10.1007/s00259-020-05029-w)
Supplement: Supplementary file 1 — (DOCX 6418 kb) [file 259_2020_5029_MOESM1_ESM.docx]

# (+)-[^18^F]Flubatine as a novel α4β2 nicotinic acetylcholine receptor PET ligand – Results of the first-in-human brain imaging application in patients with β-amyloid PET-confirmed Alzheimer’s disease and healthy controls

# EJNMMI

# Solveig Tiepolt^a#^, Georg-Alexander Becker^a#^, Stephan Wilke^a^, Diego Cecchin^b^, Michael Rullmann^a^, Philipp M. Meyer^a^, Henryk Barthel^a^, Swen Hesse^a^, Marianne Patt^a^, Julia Luthardt^a^, Gudrun Wagenknecht^c^, Bernhard Sattler^a^, Winnie Deuther-Conrad^d^, Friedrich-Alexander Ludwig^d^, Steffen Fischer^d^, Herrmann-Josef Gertz^e^, René Smits^f^, Alexander Hoepping^f^, Jörg Steinbach^g^, Peter Brust^d#^, Osama Sabri^a#^

^a^ Department of Nuclear Medicine, University of Leipzig, Liebigstraße 18, 04103 Leipzig, Germany

^b^ Department of Medicine, University-Hospital of Padova, Via Giustiniani 2, 35128 Padova, Italy

^c^ Electronic Systems (ZEA-2), Central Institute for Engineering, Electronics and Analytics, Research Centre Juelich, Wilhelm-Johnen-Straße, 52428 Juelich, Germany

^d^ Helmholtz-Zentrum Dresden-Rossendorf, Research Site Leipzig, Permoserstraße 15, 04318 Leipzig, Germany

^e^ Department of Psychiatry, University of Leipzig, Semmelweisstraße 10, 04103 Leipzig, Germany

^f^ ABX advanced biochemical compounds GmbH, Heinrich-Gläser-Straße 10, 01454 Radeberg, Germany

^g^ Helmholtz-Zentrum Dresden-Rossendorf, Bautzener Landstr. 400, 01328 Dresden, Germany

Corresponding author: Solveig Tiepolt, MD

e-mail: solveig.tiepolt@medizin.uni-leipzig.de

# Supplementary Table 1 Pearson correlation analysis between regional V*_T_*_/_f*_p_* data of (+)-[^18^F]Flubatine and injected mass in the eleven healthy controls.

| Region | |  |
| --- | --- | --- |
| Right frontal cortex | r | -.346 |
|  | p | .297 |
| Left frontal cortex | r | -.362 |
|  | p | .274 |
| Right mesiotemporal cortex | r | -.284 |
|  | p | .397 |
| Left mesiotemporal cortex | r | -.489 |
|  | p | .127 |
| Right parietal cortex | r | -.420 |
|  | p | .198 |
| Left parietal cortex | r | -.568 |
|  | p | .068 |
| Right anterior cingulate cortex | r | -.591 |
|  | p | .055 |
| Left anterior cingulate cortex | r | -.469 |
|  | p | .146 |
| Right posterior cingulate cortex | r | -.346 |
|  | p | .297 |
| Left posterior cingulate cortex | r | -.543 |
|  | p | .084 |

**Supplementary Figure 1** Typical [^11^C]Pittburgh Compound B (PiB) PET/MR images of a healthy control without evidence of Aβ plaques and a patient with mild Alzheimer’s disease (AD) with Aβ plaques in neocortical gray matter.

**Supplementary Figure 2** Exemplary T1 MR slices with all 36 volumes of interest, four unilateral regions (i.e. Corpus callosum anterior and posterior; Substantia nigra and Pons/midbrain) and 16 bilateral regions.

**Supplementary Figure 3** One-tissue compartment model (1TCM) and two-tissue compartment model (2TCM) fits (0-90 min p.i. and 0-270 min p.i.) in 36 brain regions of one healthy control (HC). PET activity measurements are given by black points. Computed total tracer amounts per cm^3^ tissue (green) are presented but in case of the 2TCM also the tracer amounts in the nondisplaceable (blue) and specific tissue compartment (red). For all brain regions the estimated total distribution volume *V*_T_ (not *V*_T_*/f*_p_) and influx rate constant *K*_1_ are given.

**Supplementary Figure 4** Box-and-whisker plots showing the regional (+)-[^18^F]Flubatine binding (V_T_*/fp*) values of the nine patients with Alzheimer’s disease and the 11 healthy controls for the bilateral a-priori defined regions, the bilateral thalamus as well as the corpus callosum posterior.

# Supplementary Figure 5 Scatter plots depicting the significant Pearson correlations between (+)-[^18^F]Flubatine binding (V_T_*/fp*) and [^11^C]PiB accumulation (standardized uptake value ratio (SUVR), reference region: cerebellar cortex) in Alzheimer’s disease (AD) patients and healthy controls (HCs).

# Supplementary Figure 6 Boxplots depicting the (+)-[^18^F]Flubatine binding (V_T_*/fp*) values with and without partial volume effect correction (PVEC) between the 9 patients with mild Alzheimer’s disease (AD) and the 10 healthy controls (HCs) in all a-priori defined regions of interest except the mesial temporal cortices which are depicted in Figure 4. Increase of the standard deviation after application of the PVE correction.

**Supplementary Figure 1**


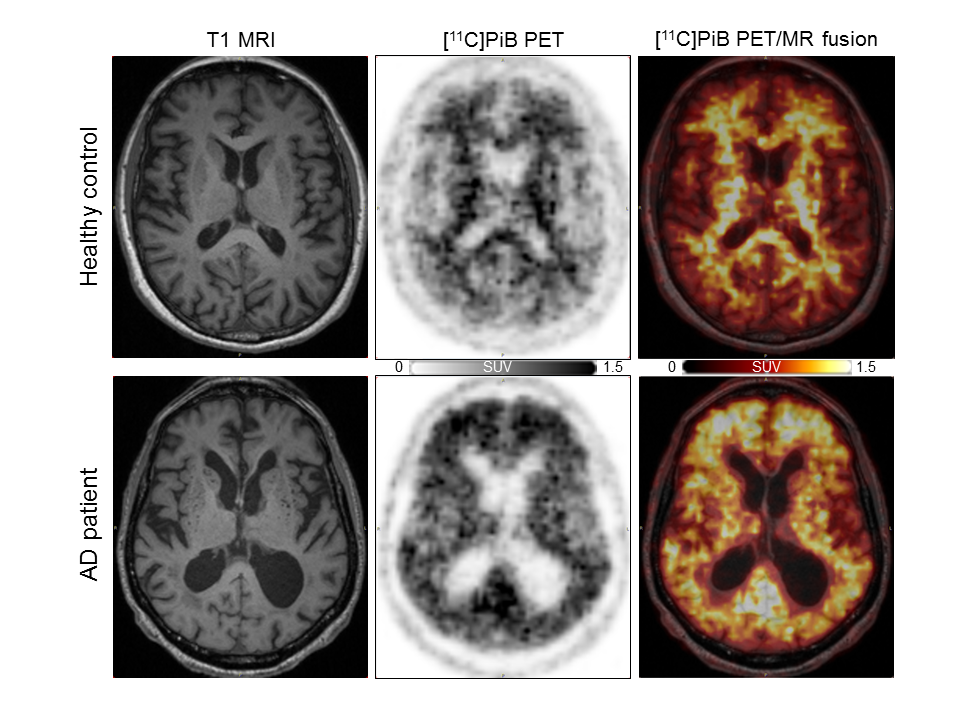


# Supplementary Figure 2


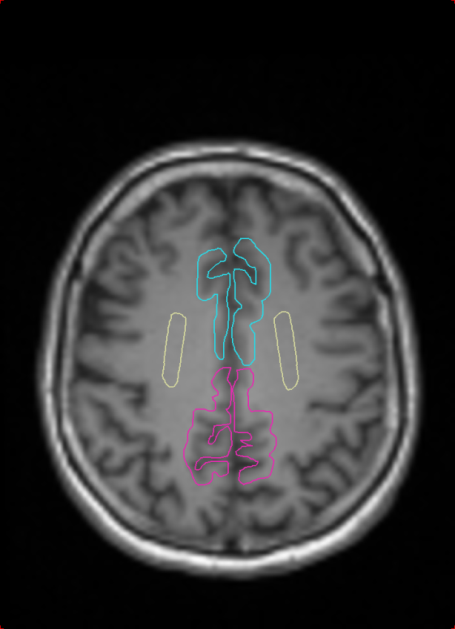

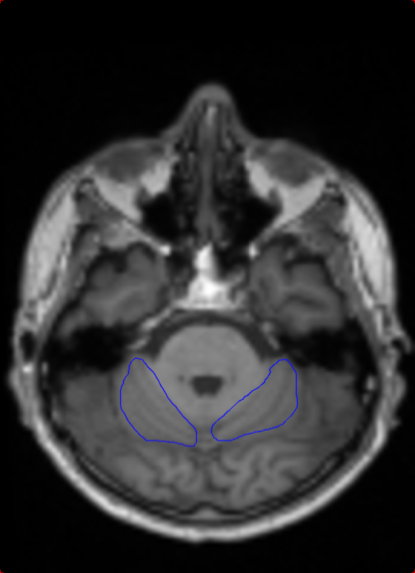

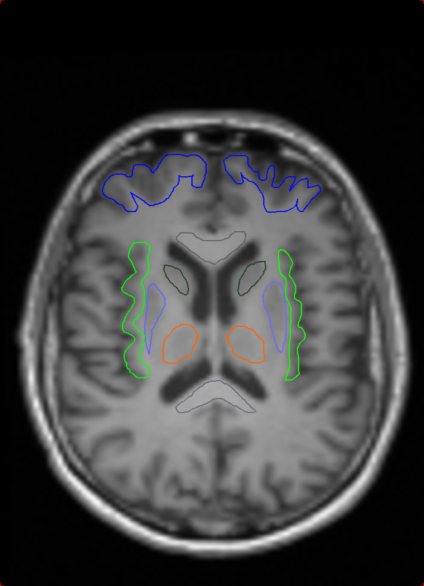

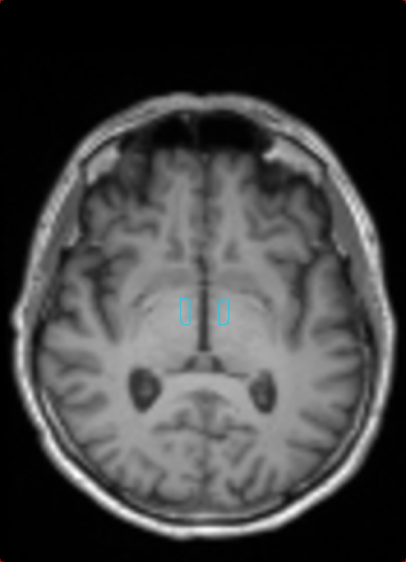

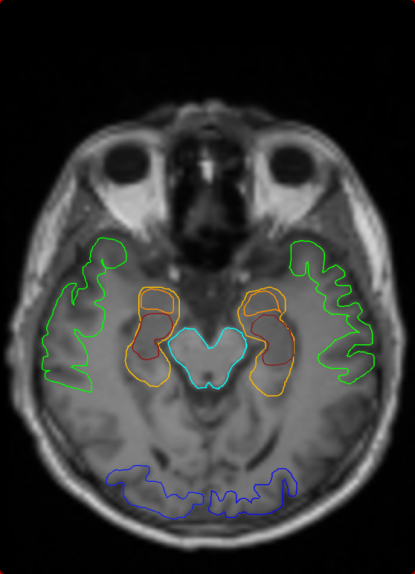

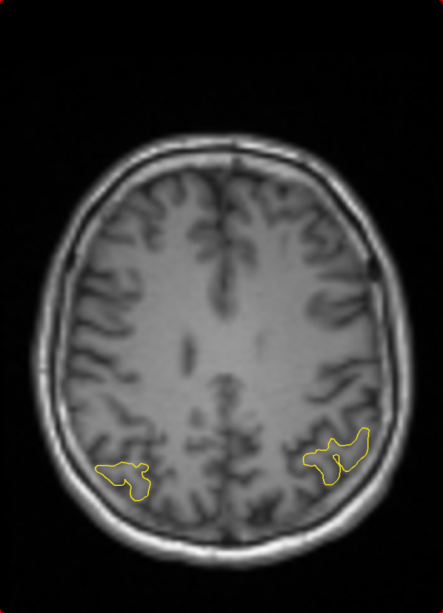

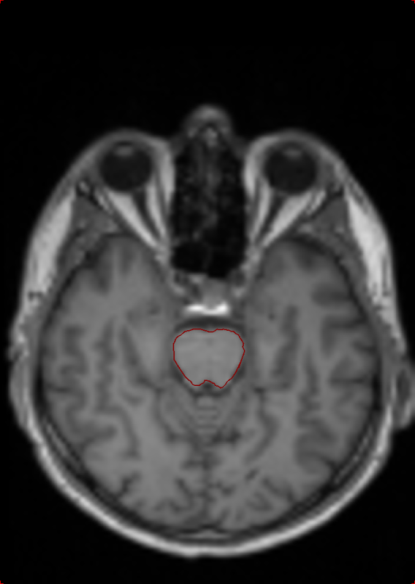


Region Legend:

1) Anterior cingulate Cortex

2) Posterior Cingulate Cortex/

precuneus

3) Centrum Semiovale

(=White Matter)

4) Parietal Cortex

5) Frontal Cortex

6) Corpus callosum anterior

7) Corpus callosum posterior

8) Head of Caudate Nucleus

9) Putamen

10) Thalamus

11) Insula

12) Hypothalamus

13) Lateral Temporal Cortex

14) Mesial Temporal Cortex

15) Amygdala

16) Hippocampus

17) Occipital Cortex

18) Midbrain/Substantia nigra

19) Pons/Midbrain

20) Cerebellar Cortex

110

1

2

3

4

5

6

7

8

9

100

120

130

140

150

160

170

190

20

180

**
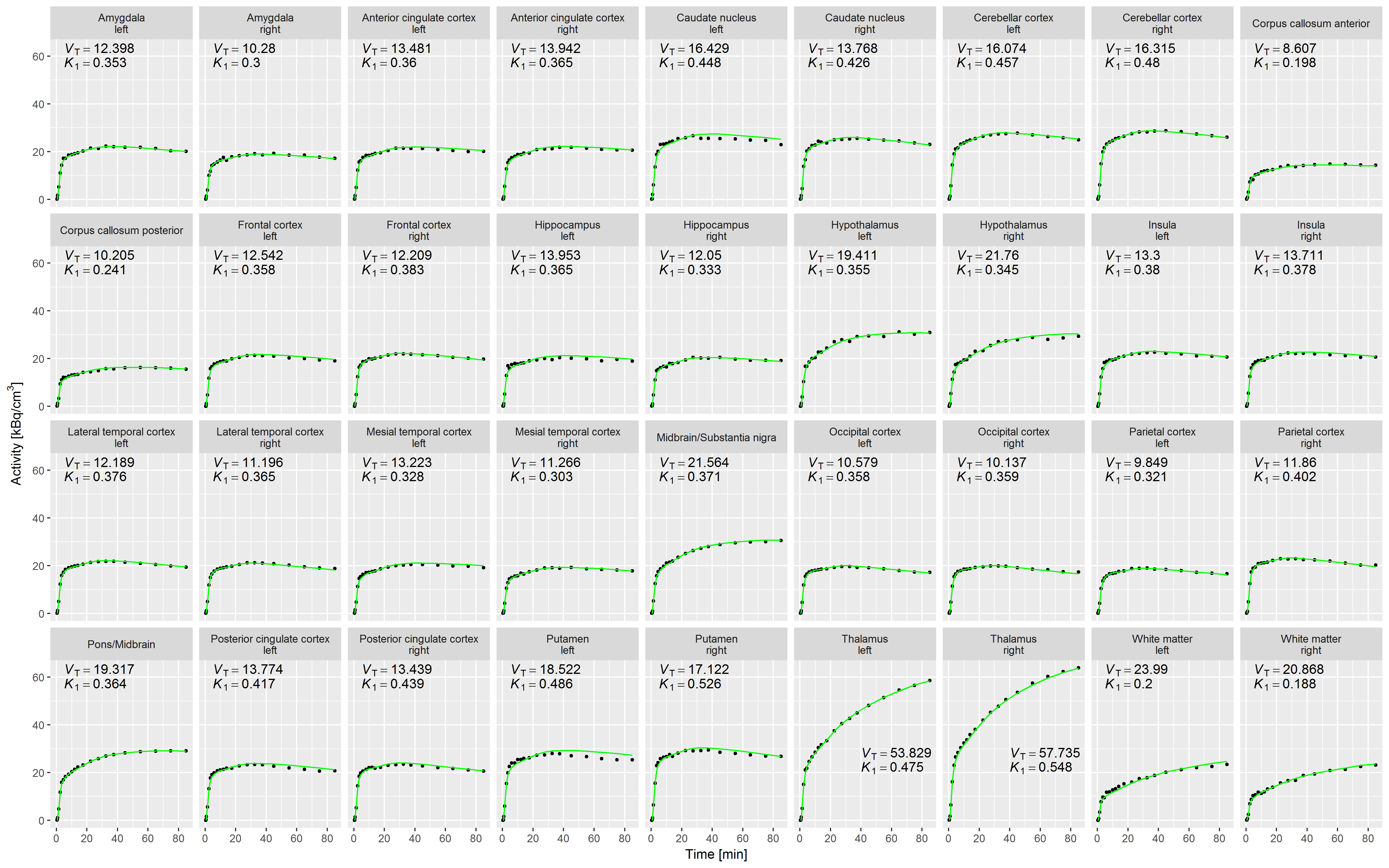
Supplementary Figure 3**


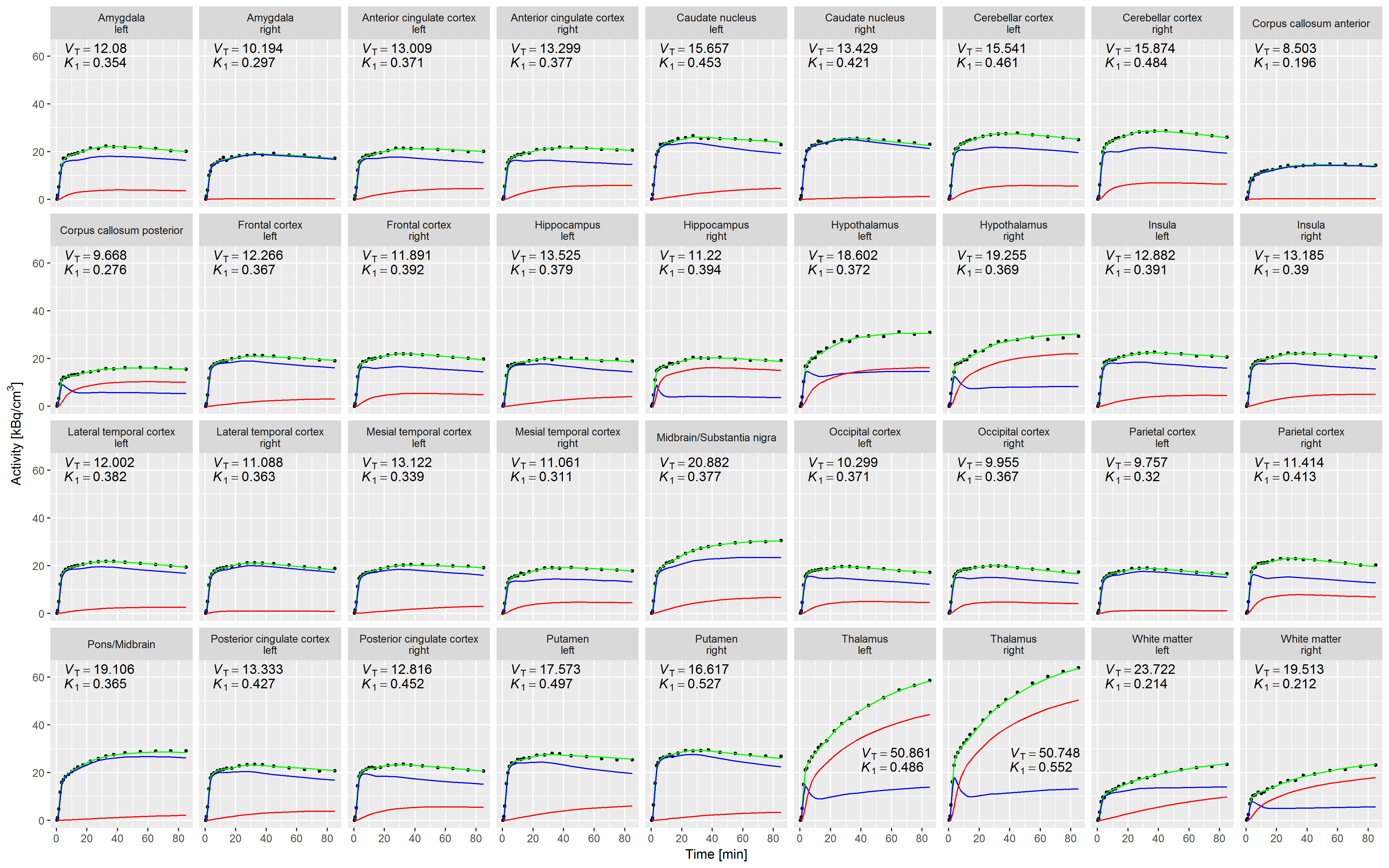


**
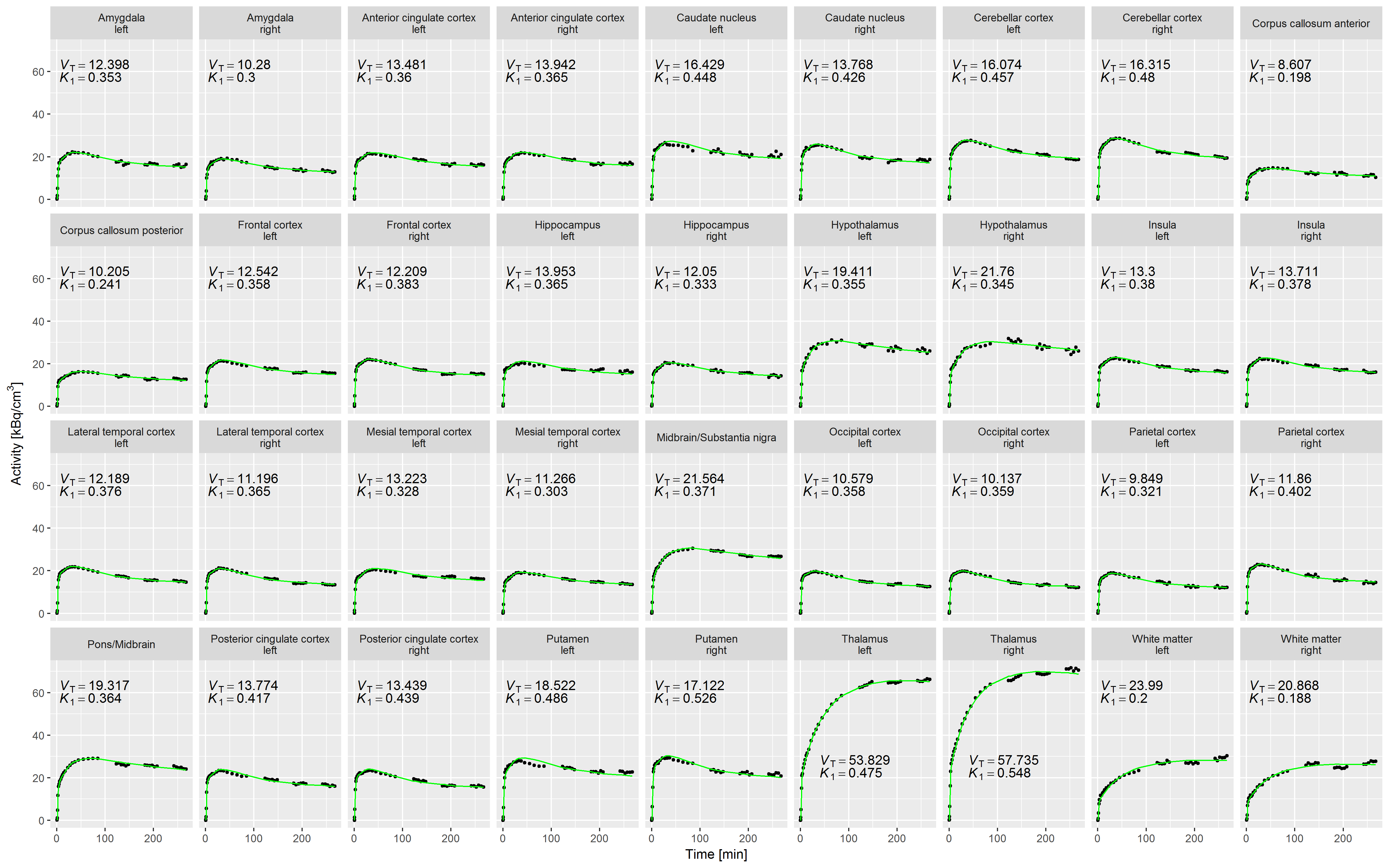
**

**
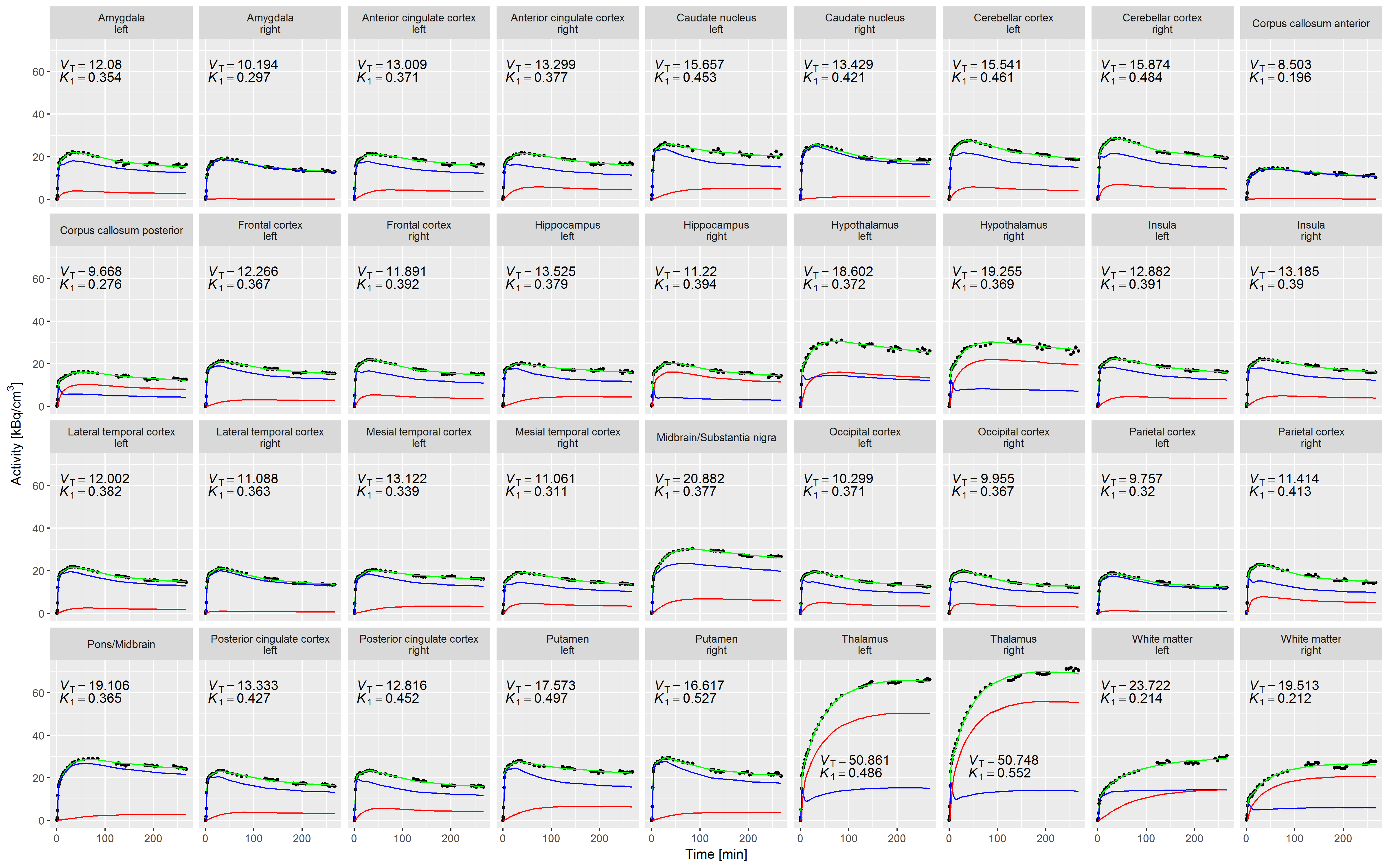
**

**Supplementary Figure 4**


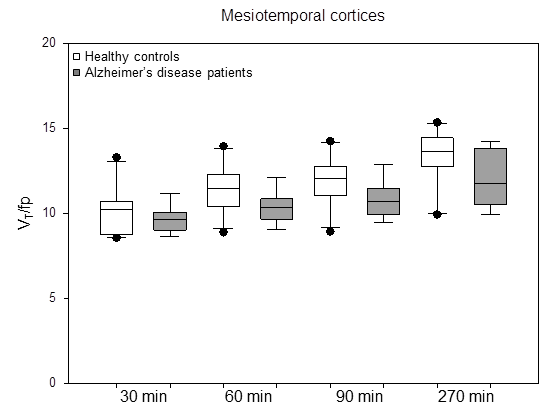

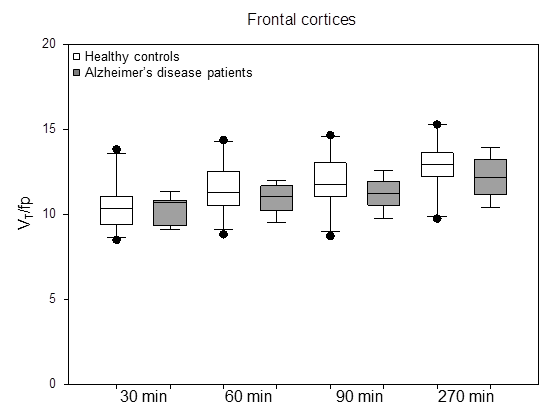

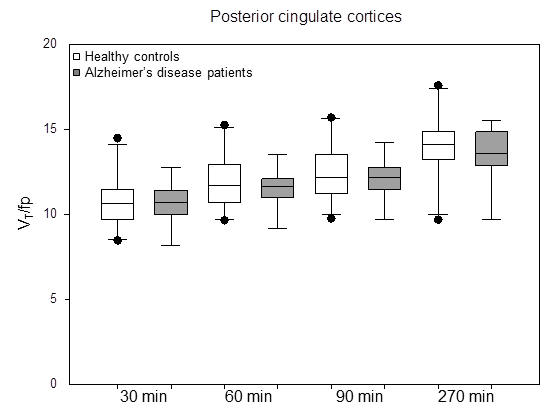

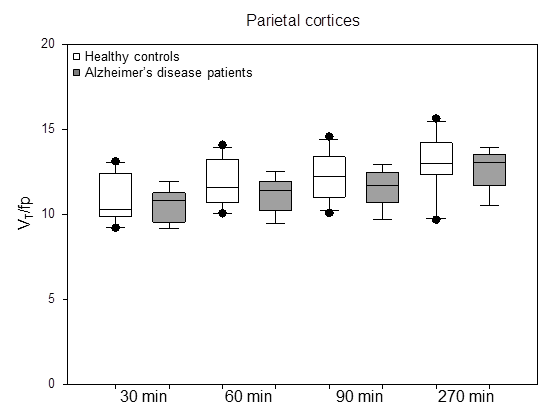

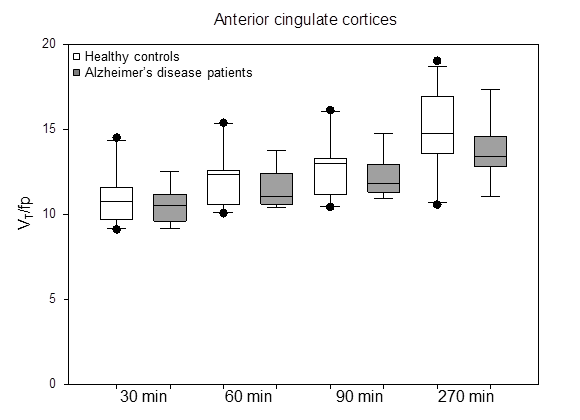

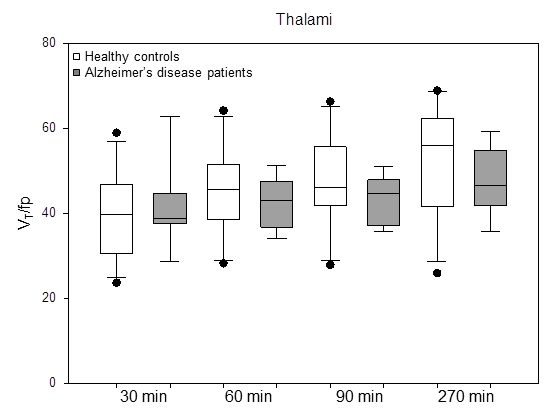

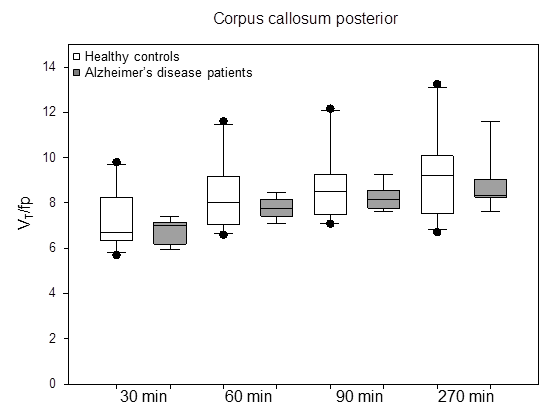


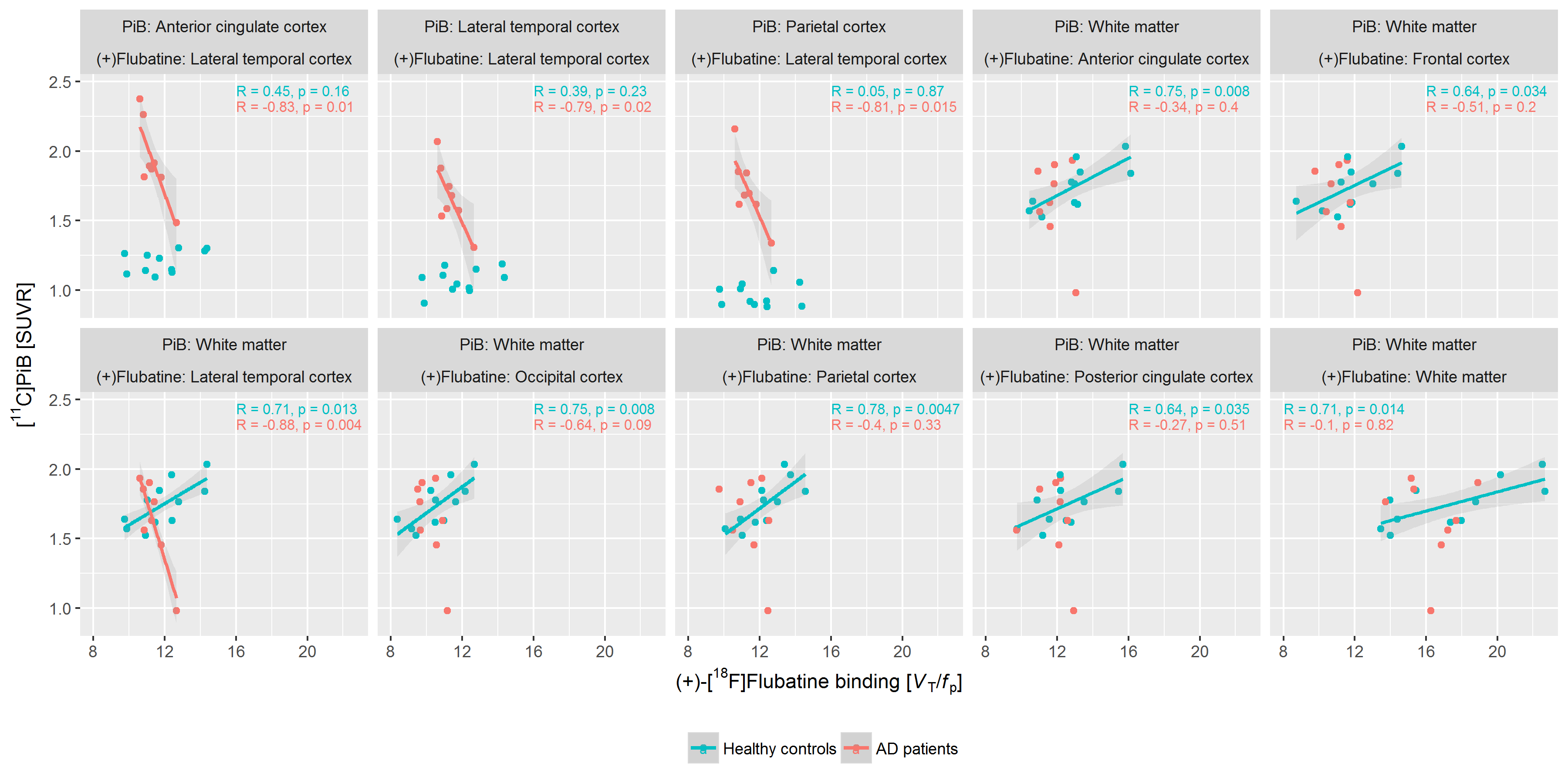
**Supplementary Figure 5**

**Supplementary Figure 6**

(+)-[^18^F]Flubatine binding (V*_T_/fp*)

(+)-[^18^F]Flubatine binding (V_T_*/fp*)

Anterior cingulate cortex right

Anterior cingulate cortex left

Posterior cingulate cortex right

Posterior cingulate cortex left

Frontal cortex right

Frontal cortex left

Parietal cortex right

Parietal cortex left

With PVEC

Without PVEC

Healthy controls

Alzheimer’s disease patients

Healthy controls

Alzheimer’s disease patients

*p*=0.247

*p*=0.134

*p*=0.340

*p*=0.132

*p*=0.088

*p*=0.226

*p*=0.176

*p*=0.254

*p*=0.188

*p*=0.353

*p*=0.113

*p*=0.329

*p*=0.239

*p*=0.453

*p*=0.339

*p*=0.399
